# Supplementary material for: Tumor-Associated Trypsin Inhibitor (TATI) as a Biomarker of Poor Prognosis in Oropharyngeal Squamous Cell Carcinoma Irrespective of HPV Status
Source: Cancers (Basel). 2021 Jun 4;13(11):2811. doi: 10.3390/cancers13112811 (PMC8200219; doi:10.3390/cancers13112811)
Supplement: Supplementary file 1 [file cancers-13-02811-s001.zip › cancers-1222353-supplementary.pdf]

cancers

MDPI

Supplemental material: Tumor-Associated Trypsin Inhibitor (TATI) as a Biomarker of Poor Prognosis in Oropharyngeal Squamous Cell Carcinoma Irrespective of HPV Status

Anni Sjöblom, Ulf-Håkan Stenman, Jaana Hagström, Lauri Jouhi, Caj Haglund, Stina Syrjänen, Petri Mattila, Antti Mäkitie and Timo Carpén

Table S1. TATI immunoscoring.

TATI Expression	TATI in Lymphocytes (Scoring)	TATI in Tumor (Scoring)	TATI in Lymphocytes (Variable in Analysis)	TATI Tumor (Variable in Analysis)
Negative	-	0	0	0
Mild positivity	+	1	1	1
Moderate positivity	++	2	2	2
Strong positivity	+++	3	3	3

Table S2. Multivariate Cox regression analysis for overall-survival (OS) and disease-specific survival (DSS) with p16 as a separate variable.

Variable	OS			DSS		
	HR	95% CI	*p*-value	HR	95% CI	*p*-value
Age	1.08	1.03–1.14	0.003 *	1.08	1.01–1.14	0.017 *
Smoking			0.029 *			0.198
Ex-smoker versus never	1.20	0.27–5.39	0.813	0.63	0.10–4.01	0.623
Current smoker versus never	4.65	1.27–17.07	0.021 *	2.62	0.62–11.08	0.189
Stage III–IV versus Stage I–II	1.75	0.73–4.20	0.209	2.22	0.76–6.47	0.146
p16– versus p16+	0.83	0.31–2.26	0.716	0.86	0.25–2.90	0.805
S-TATI	2.49	1.28–4.83	0.007 *	2.54	1.09–5.94	0.031 *

HR: Hazard ratio; CI: Confidence interval. S-TATI values are log-transformed. $p < 0.05$ *.

Cancers **2021**, *13*, 2811. https://doi.org/10.3390/cancers13112811 www.mdpi.com/journal/cancers
